# A Medical Bookstore

**Published:** 1881-12

**Authors:** 


### A Medical Bookstore.

Among the signs which mark the growth of Buffalo as a medical centre, is the opening of a bookstore on Main street, near Chippewa, devoted entirely to the sale of medical works. It is an indication, at least, that we have among us men who wish to keep abreast of the times, and whose tastes for professional study are not dwarfed by the necessities of active practice. The very presence of a good collection of books is an incentive to study, and their mute rebukes, as they stand before us, half forgotten and perhaps covered with dust, are continual inducements to mental activity. Let us, therefore, wish much success to the gentleman who has inaugurated this enterprise, and may his books not only find readers, but in a certain sense produce them.

